# Students’ initial perspectives on online learning experience in China during the COVID-19 outbreak: expanding online education for future doctors on a national scale

**DOI:** 10.1186/s12909-021-03005-y

**Published:** 2021-11-17

**Authors:** Li Li, Hongbin Wu, A’na Xie, Xiaoyang Ye, Cheng Liu, Weimin Wang

**Affiliations:** 1 Department of Research and Education, Guangzhou First People’s Hospital, South China University of Technology, Guangzhou, Guangdong China; 2 Institute of Medical Education, Peking University, Beijing, China; 3 National Center for Health Professions Education Development, Peking University, Beijing, China; 4 Institute of Medical Education, Peking University Health Science Center, No.38 XueYuan Road Haidian District, Beijing, China; 5 Princeton School of Public and International Affairs, Princeton University, Princeton, New Jersey USA; 6 Peking University Health Science Center, Beijing, China

**Keywords:** Online education, Medical education, COVID-19, China

## Abstract

**Background:**

During the early stage of COVID-19 outbreak in China, most medical undergraduate programs have to eventually embrace the maneuver of transferring to nearly 100% online-learning as a new routine for different curricula. And there is a lack of empirical evidence of effective medical education curriculum that has been completely implemented in an online format. This study summarizes medical students’ perspectives regarding online-learning experience during the COVID-19 outbreak and presents reflection on medical education.

**Methods:**

From February 21st to March 14th, 2020, the authors conducted survey of a nationally representative sample of undergraduate medical students from 90 medical schools in China. Participant demographics and responses were tabulated, and independent sample t-tests as well as multiple logistic regression models were used to assess the associations of demographic characteristics, prior online learning experience, and orientation with students’ perspectives on the online learning experience.

**Results:**

Among 118,030 medical students participated in the survey (response rate 52.4%), 99,559 provided valid data for the analysis. The sample is fairly nationally representative. 65.7% (65,389/99,559) supported great orientation and 62.1% (61,818/99,559) reported that they were satisfied with the ongoing online-learning experience. The most common problem students would encounter was the network congestion (76,277/99,559; 76.6%). Demographics, learning phases, and academic performance were associated with online-learning engagement and perceptions. Formal orientation and prior PU (perceived usefulness of online learning) were significantly positively associated with the satisfaction and evaluation of the online learning experience (*p* <  0.001).

**Conclusions:**

Data from this national survey indicates a relatively positive role of online learning as a formal teaching/learning approach in medical education. Considerations should be made regarding such application in aspects of students’ different learning phases. We suggest that further policy interventions should be taken from technological, organizational, environmental, as well as individual aspects, to help improve the outcome of online learning for future doctors.

## Background

Caused by SARS-CoV-2, the 2019 novel coronavirus disease (COVID-19), first reported in Wuhan, China, has rapidly resulted in a global pandemic [1]. Since the COVID-19 outbreak, a large number of healthcare resources have been allocated to dealing with the outbreak [2]. Medical education and healthcare training were often greatly affected during previous infectious disease pandemics [3]. The COVID-19 has also transitioned the education and training of medical students and healthcare workers (HCWs) [4]. Most, if not all, of the undergraduate medical education and training programs, have been put on halt since the outbreak, especially during the initial stage of the outbreak. Across the world, in-person medical classes have been immediately cancelled; meanwhile, online and other technology-enhanced learning is considered as an alternative innovation in academic medicine during the global educational crisis [5–7]. However, little is known about how to provide future doctors with effective online education on an unprecedentedly large scale. In this study, using survey data of about 25% of Chinese undergraduate medical students during the COVID-19 outbreak, we collected one of the first evidence on student engagement and responses to online education.

In response to the outbreak, the Ministry of Education (MOE) of the People’s Republic of China immediately issued interim guidance of instructions on deploying online-learning for the higher education institutions during the COVID-2019 outbreak in February [8–10]. Since then, over 7,133,000 online-learning sessions covering more than 942,000 virtual courses have taken place within 2 months [11]. As a critical component of higher education, medical education during the COVID-19 received particular policy attention in China as it is a large practice-based discipline with many sub-specialties and many of its learning activities were formerly arranged to take place in clinical settings. Moreover, instructors in medical schools are also HCWs, who may be working in the frontline to fight against COVID-19. Online learning actions have then been taken by medical universities and colleges around China as responses: contacting students/trainees regarding the latest learning schedule and performing needs assessment, preparations made at least 2 weeks before the initiation of online-learning programs based on available resources, and development of new teaching and learning programs to meet the emerging needs. While centrally planned by the MOE, these online distant learning programs, nevertheless, vary across regions and schools in target audiences, contents, and delivery formats.

Across the globe, online education has gradually become an integrated part of modern medical education [12, 13]. As the COVID-19 situation was initially expected to last for months globally, and later expand more than 1 year, lots of medical undergraduate programs must embrace the maneuver of transferring from the traditional, mostly in-person, training approach, to online-learning or blending learning as a new routine for different curricula. Concerns have been quickly drawn to the effectiveness and quality of these programs, compared with traditional face-to-face classroom or bedside teaching and learning.

However, there is relatively little empirical evidence of successful medical education curriculum that has been completely implemented in an online format, despite sporadic reports on exploratory attempts of blended learning approaches by combining internal network platforms as aids. In order to ameliorate the understanding of such learning experience, especially in regard to how students are conceiving such an approach, we conducted a nationwide survey on students’ initial perspectives regarding online-learning experience during the COVID-19 outbreak among undergraduate medical students in China. In this study, we summarize and reflect on the current practices of formal online teaching and learning.

## Method

### Survey instrument

We, the authors, represent both the National Centre for Health Professionals Education Development (NCHPED), a research institution established at Peking University and jointly commissioned by Chinese MOE and National Health Commission (NHC) in order to better utilize the resources on medical education on the national level and to promote the reform and further development of health professionals education in China, and the National Ministry of Education Advisory Council for Teaching and Learning in Clinical Medicine, an expert panel providing guidance and make critical decisions regarding clinical medical education in China. We generated the survey instrument based on official reports from the medical higher education institutions in China, which include a varying arrangement of online learning and instruction schedules. After the initial design, we consulted a panel of experts in clinical practices, medical education, and education policy, and performed multiple pilot runs of the survey among random groups of undergraduate medical students in China to ensure the reliability and validity of the survey instrument. The final survey text is available upon request. Items of the survey instrument can be categorized into three domains: 1) basic information and demographic characteristics, including gender, the area of their home location (rural or urban), learning phases, and self-evaluated academic performances in percentile (top 25, 25–50%, 50–75%, bottom 25%), 2) students’ previous online-learning experience, 3) students’ ongoing online-learning experience and its evaluation during COVID-19. Regarding the undergraduate clinical medical training phases, we intentionally divided it into four learning phases (general education, basic medical education, clinical medical education, and clerkship rotation), which is well-accepted and previously recorded [14].

### Sample and participants

We sampled 90 medical universities out of 181 in China who were able to transit to online learning for medical students, and had announced their online learning schedules, as well as agreed to participate in the survey after reaching consent. Between February 21st, 2020, and March 14th, 2020, we distributed the questionnaire to medical students in these 90 medical universities (total enrolled undergraduate medical students *n* = 225,329) via the professional version of a popular online survey platform WJX (https://www.wjx.cn/). University administrators made invitations to each student and students voluntarily participated in the survey. The survey was distributed to each institution 1 week after its start date of the new “online” semester to ensure that have had experienced in various online courses their institutions had offered. Responses were anonymous but each survey was unique to that study participant and could not be shared or completed more than once. Only survey responses from medical students (clinical medicine undergraduates, who will eventually obtain their Bachelor of Medicine Degree) will be included for further analysis.

### Study measures

#### Prior online learning experience

Based on the Technology Acceptance Model (TAM) and previous empirical research [15], we investigated students’ online learning experience: perceived usefulness (PU) of online learning. We utilized a question to investigate students’ PU of these modes according to their prior experience.

#### Current online-learning experience

Students’ current formal online learning experiences were probed from 3 areas. First, general evaluation of the formal orientation (the approach provided by institutions and teachers/instructors to help students get familiar with the learning platform/software, and contents and learning arrangements) process on how to proceed with online learning provided by the institutions to medical students is measured by five-point anchors of “excellent, good, neutral, poor, bad” and an extra anchor of “not clear”. Second, students report problem(s) that they have encountered during online learning. Building on prior research, the survey provided multiple possible difficulties or problems for students to choose from, including disliking online-learning in general, network congestion, poor arrangements and scheduling, instructors prepared for courses poorly, untimely feedback and answering questions, insufficient interaction, and insufficient learning resources. Students could also provide additional answers regarding the question. Third, students also provide their levels of satisfaction with current online learning experiences using a five-point Likert Scale (strongly satisfied, satisfied, neutral, dissatisfied, or strongly dissatisfied). We are interested in students’ responses to online medical education during the special COVID-19 times, which is the first of its kind to be implemented as a formal learning approach at a national (or, global) scale.

### Statistical analysis

First, we tabulated and summarized demographic characteristics for our study sample. Next, we examined responses to each measure of current online learning experiences. When investigating the effect of formal orientation on how to proceed with online learning, responses of evaluation regarding formal orientation were collapsed into two categories with the values of 1 and 0 respectively: good orientation (excellent and good) and poor orientation (neutral, poor, bad, and no idea). Independent sample t-tests were used to explore its association with whether students would encounter various difficulties, students’ level of satisfaction (satisfied and extremely satisfied) towards current online-learning. As a measure of effect size, considering that *p*-values are affected by sample size in t-tests, we calculated the effect size of Cohen’s d for each t-test to help interpret the importance of differences observed (with 0.2 indicating a small effect, 0.5 a medium effect and 0.8 a large effect) [16].

Finally, we used multiple logistic regression models to assess independent associations between hypothesized predictors (gender, learning phases, student location, academic performances, prior online-learning experience, and perception regarding the usefulness of formal orientation for online-learning provide by institutions) and each statement of the evaluation regarding the current online-learning experience, as well as the level of satisfaction towards online-learning. The statistical analysis was performed using STATA 14© (College Station, TX, USA) with *p* <  0.05 defined as statistically significant.

## Results

### Demographic characteristics

Of the 225,329 medical undergraduate students we asked to participate, 118,030 responded (52.4%). We collected data to guarantee its quality in terms of response time, medical school name and start time of online education. After data cleaning, the sample consisted of 99,559 medical students from 90 medical schools. The final sample accounted for 24.4% of all medical undergraduate students (clinical medicine undergraduates) in China (408,764 in total). Compared with the national data, the sample data showed a relatively high proportion of female, urban and medical students from comprehensive universities. But overall, the sample is fairly nationally representative (Table 1).

**Table 1:** Demographic Characteristics of 99,559 Participants at 90 Medical Schools compared with national medical student demographics, from Chinese Medical Student Survey ^a^ and China Working Committee for the Accreditation of Medical Education (WCAME) ^b^

	Participants, no (%)	Nationally, %
Gender		
Female	60,815 (61.1)	55.0^a^
Male	38,744 (38.9)	45.0^a^
Learning Phases		
General education	14,774 (14.8)	–
Basic medical education	43,026 (43.2)	–
Clinical medical education	30,869 (31.01)	–
Clerkship rotation	10,890 (11.0)	–
Student location ^c^		
Rural	41,538 (41.7)	36.9^a^
Urban	58,021 (58.3)	63.1^a^
Academic performance		
Top 25%	36,440 (36.6)	–
25–50%	35,687 (35.8)	–
50–75%	21,112 (21.2)	–
Bottom 25%	63, 20 (6.4)	–
PU regarding online learning based on prior experience		
Not Useful	68,421 (69.6)	–
Useful	31,138 (31.4)	–
Medical school type ^d^		
Free-standing	58,982 (59.2)	61.6^b^
Comprehensive	40,577 (40.78)	38.4^b^
Region		
East	38,134 (38.3)	38.9^b^
Middle	41,341 (41.5)	35.8^b^
West	20,084 (20.2)	25.4^b^

*Abbreviation*: *PU* perceived usefulness

^a^ Data Source: 2019 China Medical Student Survey (CMSS), conduct by National Centre for Health Professionals Education Development (NCHPED), authorized by Ministry of Education (MOE) and National Health Commission (NHC) [38]

^b^ Data Source: surveillance data from Higher Education Evaluation Center of MOE in 2018, medical portion provided to NCHPED. Certain national data are unavailable to the public

^c^ China is of typical “urban-rural dual structure”, and we asked the survey participants to select this information based on their permanent residency record. While the outbreak of COVID-19 in China was around the time of Spring Festival, the survey participants are in their home area, and since the travel ban, they had to carry out online learning at home when medical universities resumed learning with online approaches

^d^ Free-stand medical school and medical school in comprehensive universities are the types available in China [39]

### Students’ self-reported opinion regarding online learning

Nearly 2/3 (65.7%) of the respondents agreed that their institutions had provided good formal orientation before the launch of formal online learning, with 19,586/99,559 (19.7%) selected “excellent” and 45,803/99,559 (46.0%) selected “good”. Only a small portion of the surveyed students thought that such orientation is poor (2958/99,559; 3.0%) or bad (1575/99,559; 1.6%).

The two most common problems students would encounter during the first week of online learning were network congestion (76,277/99,559; 76.6%) and insufficient interaction (44,602/99,559; 44.8%). Relatively, a minority of students reported having experienced instructors’ poor preparation of courses (6356/99,559;6.4%), untimely feedback and question-answering from instructors (9657/99,559; 9.7%). 62.1% (61,818/99,559) of the students were satisfied (satisfied and extremely satisfied) with the current online-learning experience, 8.7% (8650/99,559) of the students were not satisfied (dissatisfied and extremely dissatisfied), whilst another 29.2% (29,091/99,559) of responded students were neutral regarding how satisfied they feel about such experiences (Table 2).

**Table 2:** Self-reported opinions towards online learning among 99,559 participants in 90 medical schools

***Formal orientation provided by institutions***	No. (%) ^a^				
	Excellent	Good	Neutral	Poor	Bad
Students’ opinion ^b^	19,586 (19.7)	45,803 (46.0)	28,251 (28.4)	2958 (3.0)	1575 (1.6)
***Difficulties/problems***	Not Encountered		Encountered		
Rejection of online teaching	77,650 (78.0)		21,909 (22.0)		
Network congestion	23,282 (23.4)		76,277 (76.6)		
Poor arrangements and scheduling	78,885 (79.2)		20,674 (20.8)		
Poor preparation of courses	93,203 (93.6)		6356 (6.4)		
Untimely feedback & question-answering	89,902 (90.3)		9657 (9.7)		
Insufficient interaction	54,957 (55.2)		44,602 (44.8)		
Insufficient learning resources	70,711 (71.0)		28,848 (29.0)		
***Level of satisfaction***	Extremely Satisfied	Satisfied	Neutral	Dissatisfied	Extremely Dissatisfied
Overall satisfaction towards current online-learning experience	30,721 (30.9)	31,097 (31.2)	29,091 (29.2)	5992 (6.0)	2658 (2.7)

^a^ Percentages may not add to 100 because of rounding

^b^ Number (1386) and percentage (1.4%) of students selected “not clear” was not shown in the table, so the percentage may not add to 100

### The effect of institutional formal orientation of online learning on students’ online learning experiences

We further investigated the possible associations between orientation the students received from their learning institutions prior to the start of their online courses and students’ encountering difficulties/problems during online learning, as well as their overall satisfaction. Results are shown in Table 3. Students who claimed to have received poor formal orientation from institutions were more likely to report that they’ve encountered difficulties/problems, while the item Network Congestion has the smallest effect (Cohen’d = 0.08).

**Table 3:** T-tests and the effect size of orientation towards online-learning

Student’s view towards formal orientation provided by institutions	Good ^a^		Poor		Diff	*p*-value	Cohen’s *d* ^b^
	mean	95% CI	mean	95% CI			
***Difficulties/problems encountered in online learning***							
Rejection of online teaching	0.16	0.15–0.16	0.34	0.33–0.34	0.18	< 0.001	0.43
Network congestion	0.78	0.77–0.78	0.75	0.74–0.75	−0.03	< 0.001	0.08
Poor arrangements and scheduling	0.15	0.14–0.15	0.33	0.32–0.33	0.18	< 0.001	0.44
Poor preparation of courses	0.04	0.03–0.04	0.12	0.11–0.12	0.08	< 0.001	0.33
Untimely feedback & question-answering	0.08	0.07–0.08	0.14	0.13–0.14	0.06	< 0.001	0.20
Insufficient interaction	0.41	0.41–0.42	0.52	0.50–0.51	0.11	< 0.001	0.21
Insufficient learning resources	0.25	0.24–0.25	0.37	0.36–0.37	0.12	< 0.001	0.27
**Overall satisfaction towards online-learning experience** ^***c***^	0.79	0.79–0.80	0.29	0.29–0.30	−0.50	< 0.001	1.04

^a^ Responses of evaluation regarding “formal orientation” were collapsed into two categories: “good formal orientation” (excellent and good) and “poor formal orientation” (neutral, poor, bad, and no idea), and given relative value of 1 and 0 respectively

^b^ As a measure of effect size, Cohen’s *d* was calculated (with 0.2 indicating a small effect, 0.5 a medium effect and 0.8 a large effect)

^c^ Selection of “Agree” and “strongly agree” were considered for the overall satisfaction

Participants perceiving poor institutional formal orientation tend to respond higher proportion of problems during online learning, including rejection of online teaching (0.34 vs 0.16, Diff 0.18, *p* <  0.001), poor arrangements and scheduling (0.33 vs 0.15, Diff 0.18, *p* <  0.001), poor preparation of courses (0.12 vs 0.04, Diff 0.08, *p* <  0.001), untimely feedback and question-answering (0.14 vs 0.08, Diff 0.06, *p* <  0.001), and insufficient learning resource (0.37 vs 0.25, Diff 0.12, *p* <  0.001). However, the “formal orientation” showed little effect regarding students’ encountering network congestion problems (0.75 vs 0.78, Diff − 0.03, *p* <  0.001). The satisfaction of online learning experience is also significantly positively associated with the perception of prior orientation towards online learning (good formal orientation 0.79 vs. poor formal orientation 0.29, Diff = 0.50, *p* <  0.001), which has the largest effect (Cohen’d = 1.04).

### Multiple regression models

Among the various potential problems we have probed in the survey, *network congestion* and *insufficient interaction* between instructors and peers were the most common ones. We included these two problems together with the online-learning satisfaction as dependent variables and reported the results of multiple logistic regression analysis in Table 4. Compared with students in general education phase, students in clerkship rotation phase (OR = 0.35; 95% CI: 0.33, 0.37) and clinical medical education phase (OR = 0.53; 95% CI: 0.50, 0.56) were less likely to report of having network problems during online learning. In addition, compared with students in general education, those in their clerkship rotation (OR = 1.43; 95% CI: 1.36, 1.51) and clinical medical education (OR = 1.18; 95% CI: 0.14, 1.23) tend to be more concerned about insufficient interaction during online learning. Interestingly, compared with students in the top 25% in their academic performances, students ranked 25–50% (OR = 0.91; 95% CI: 0.88, 0.93), 50–75% (OR = 0.85; 95% CI: 0.82, 0.88), and bottom 25% (OR = 0.75; 95% CI: 0.71, 0.79) tend to be less likely concerned about interaction problem during online-learning. Students located in urban areas (OR = 0.79; 95% CI: 0.77, 0.82) were also less likely to report about network congestion problems compared with students located in rural areas. Male students (OR = 0.82; 95% CI: 0.82, 0.87) tend to report less about network congestion compared to female students but were more likely to be concerned about insufficient interaction during online learning (OR = 1.17; 95% CI: 1.14, 1.20).

**Table 4:** Adjusted Odds of Agreement with the Evaluation of Online Education among 99,559 Participants at 90 Medical Schools ^a^

	OR (95% CI) ^b^		
Characteristic	Network Congestion	Insufficient interaction	Online-learning Satisfaction
**Gender**			
Female (reference)	1.00	1.00	1.00
Male	0.84(0.82,0.87) ^d^	1.17(1.14,1.20) ^d^	0.96(0.93,0.99) ^c^
**Learning Phases**			
General education (reference)	1.00	1.00	1.00
Basic medical education	0.86(0.82,0.91) ^d^	1.03(0.99,1.08)	0.93(0.89,0.98) ^c^
Clinical medical education	0.53(0.50,0.56) ^d^	1.18(1.14,1.23) ^d^	0.75(0.72,0.79) ^d^
Clerkship rotation	0.35(0.33,0.37) ^d^	1.43(1.36,1.51) ^d^	0.67(0.63,0.71) ^d^
**Student location**			
Rural(reference)	1.00	1.00	1.00
Urban	0.79(0.77,0.82) ^d^	0.96(0.94,0.99) ^c^	1.08(1.05,1.12) ^d^
**Academic performance**			
Top 25%(reference)	1.00	1.00	1.00
25–50%	1.05(1.01,1.09) ^c^	0.91(0.88,0.93) ^d^	1.04(1.00,1.08) ^c^
50–75%	1.06(1.02,1.11) ^c^	0.85(0.82,0.88) ^d^	1.05(1.01,1.09) ^c^
Bottom25%	1.04(0.98.1.12)	0.75(0.71,0.79) ^d^	0.96(0.90,1.02)
**Prior PU**	0.91(0.88,0.94) ^d^	0.75(0.73,0.77) ^d^	2.05(1.98,2.13) ^d^
**Orientation is Good**	1.24(1.20,1.29) ^d^	0.73(0.71,0.75) ^d^	7.67(7.43,7.91) ^d^

*Abbreviation*: *PU* perceived usefulness

^a^ Logistic regression models were run for agreement with each statement of the evaluation on the online education to estimate associates for the following factors: sex, learning phases, student location, academic performance, perceived usefulness regarding prior online learning and orientation for the online learning from institutions. To control for differences between institutions, all the models we used the institutions fixed effects (not reported) and we clustered standard errors by institutions

^b^ Odds ratio (95% confidence interval)

^c^ *P* < .05 (*P* values not shown)

^d^ *P* < .001 (*P* values not shown)

Holing all else factors equal, students in clinical medical education phase (OR = 0.75; 95% CI: 0.72, 0.79) and clerkship rotation phase (OR = 0.67; 95% CI: 0.63, 0.71), compared with students in general education phase seemed less satisfied with online learning. Compared with female students, male sex was also less likely to be satisfied with the online-learning experience (OR = 0.96; 95% CI: 0.94, 0.99). Students who thought that formal orientation provided by their institutions were good (OR = 7.67; 95% CI: 7.43, 7.91) were significantly more likely to be satisfied with the online-learning format compared with those thought such orientation were poor. Students who found the previous online-learning experience useful (OR = 2.05; 95% CI: 1.98, 2.13) were more likely to be satisfied with the current online-learning compared with those who didn’t find it useful.

## Discussion

To our knowledge, this study involving over 99,000 participants from 90 medical schools geographically dispersed in China is the first nationally representative survey of Chinese medical undergraduate students’ perspectives on formal online learning. We provide an overview illustration of current online medical education approaches implemented in China in response to the COVID-19 outbreak (and other potential pandemics).

Our study demonstrated that the majority of responding medical students were satisfied with the online learning program provided by their institution (62.1%), indicating the general acceptance among students regarding online-learning approach aimed for undergraduate medical education. Previously conceived as an add-on, online learning was rarely suggested as feasibly expanding in the existing medical educational system [17]. Research projects trying to address such issue often face an ethical dilemma concerning educational equity and feasibly in design [18–20]. However, given the actual situation of the COVID-19 outbreak that impedes the traditional face-to-face teaching and learning, online learning serves as a first-line solution for medical education. Our study supports the assumption of using online learning as one of the feasible options of learning approaches in medical education.

Furthermore, we have revealed that students’ online-learning experiences were affected in multiple contexts, including technology, organizational commitment, the situated physical and social environment, as well as individual characteristics.

### Technological context

As discussed above, two of the most significant challenges (or barriers) among students participating in online learning sessions are network congestion (76.6%) and insufficient interaction (44.8%). These two, which fall in technological context, are also the most common issues that most online learning would encounter, especially concerning disciplines that have much emphasis on hands-on practices. Internet connection serves as a critical infrastructural component to e-learning or mobile learning approaches [21, 22]. With current technology advancements, people often underestimate the possibility of network congestion, especially when a large number of participants are using the same internet services at the same time. Students at the beginner level of their learning phases would experience their learning activities in a more knowledge-based manner. Didactic learning formats usually suffice at this stage; and, in the general context of disease outbreak, such learning activities are more internet-dependent, compared with learning activities among advanced stages students would encounter. Internet connection issues thus become more apparent towards beginner phase students.

Notably, students’ satisfaction regarding online learning decreases as students move to more advanced learning phases, indicating students’ perception of online learning might be content-specific. As medical students proceed to an advanced learning stage, most of the learning contents require students to demonstrate psychomotor skills and behaviors, such as performing clinical procedures. Such contents are still considered difficult to be integrated with an online format or virtually, albeit endeavors made to establish platforms providing high-fidelity virtual learning experience (such as the national iLAB-X project). It is notable that certain attempts have been made to address the disadvantages online learning is facing towards students developing psychomotor or behavioral skills, including inviting students’ family member to act as standardized patients during students’ learning about performing history taking and physical examinations, using accessible material in home environment for procedural skill exercises (i.e. grapefruit skin as task trainers for tying surgical knots), and online live discussion on drawing concept-maps for clinical reasoning instructions. As of the stage upon completion of data analysis of this survey, certain technology in the various online learning platforms, public (e.g. ZOOM or Tencent Meeting) or private, have not yet included functions such as providing virtual breakout rooms, or supporting remote ward rounds in the bed-side teaching setting.

### Organizational context

The organizational context of a technology-integrated system refers to the characteristics and resources, including linking structures, internal communication processes, institution size, and the number of slack resources [23]. In online medical education, preparation from the faculty side is expected from students, which involves instructional design specifically targeting students’ online-learning needs and aligning with overall curriculum requirements. Approaches, such as choosing appropriate learning platforms based on available resources or developing new resources, would provide students a sense of freedom in selecting adequate resources to adapt their own learning needs. Meanwhile, determining instruction, student facilitation, and interaction strategies would allow students to perceive the participating learning experiences were being arranged with care [24]. As students were not expected to be ubiquitously familiar with most of the online learning platforms, formal orientation to how to get the best out of online learning should also be an integral part of the online learning preparation and implementation. Role of feedback towards students that lead to effective learning (even during online learning) has been universally acknowledged [22, 25–27]. However, instructors’ competence in providing constructive feedback is somewhat unsatisfactory. Such an issue could be even more outstanding when learning activities are taking place online as communication processes are limited, whilst students still expect effective and timely feedback. Feedback very often occurs during teacher-learner or peer-to-peer interaction, as well as formal feedback activities.

These intentional faculty good practices, which trained faculty members are more likely to exhibit, require organizational commitment, and could be transferable towards online instruction, is demanded if such awareness of providing faculty development opportunities is expected. Institutions could provide interim guidance and tips on how to improve online teaching activities, allowing try-outs or rehearsals from faculty before classes with a large audience, providing mandated formal orientation upon launch of various online course, monitoring learning activities, encouraging good feedback behaviors from teachers, and even perform immediate trouble-shooting or having a backup plan for the course delivery once faculty encountered technical problems [22]. Such approaches call for measures taken on a systematic level within the institution and setting up a community of practice in online-learning both within and outside the institution may also help improve and expand the significance of effective online-instruction good practices [28–31].

### Environmental context

The environmental context usually referred to the physical and social environment one technology-imbued institution is located in, including the general degree of development for certain technology, the presence or absence of technology service providers, and the regulatory environment [23]. As online learning is a widely accepted, although not mandated, approach to aid the existing medical education programs, most students have exposed to online learning formats with experiences regarding different online learning formats, both formal and informal ones. At the national level, both MOE-lead and NHC-led platforms with different contents are already available before the disease outbreak. At the institutional level, universities also have their self-maintained online learning services for students. However, the determinant affecting students’ perspective towards online learning during this specific circumstance may possibly be what terminal platforms the students are actually using. Unlike previous circumstances, where students could have easy access towards computers or internet services designated for educational purposes, currently students can only access through their own devices (personal computers or mobile phones) and via home internet. Students located in rural areas are more likely to be affected by internet issues and may be less preferable towards such alteration in learning. Rural or other resource-constrained areas may benefit less from online learning.

### Individual aspect

It is worth the attention that our study does not include the investigation regarding the impact of individual background difference on their perception. In compliance with adult learning theory [32] and consistent with previous research correlating experience and user intention proposed [14, 33], our finding suggests a strong association between the prior online learning experience and satisfaction towards the current mandated online formal learning. Good prior PU from students tends to lead to significantly increased satisfaction towards their upcoming online learning activities, possibly implying that awareness should be raised towards providing enjoyable and engaging online learning content and formats to our students from the very beginning of their medical education journey. Special attention should be paid towards students who showed early signs of being not able to adapt or rejecting towards online learning. These students who may have negative experience with online learning should be provided with extra guidance to better accept such format. Aligning with previous research showing a negative correlation between learning performance and satisfaction with online courses [34], our results also indicate that students’ personal academic performances may also have an impact on their perception towards certain elements of the online learning experience: students with better academic performances may care more about heuristic interactions, which could possibly meet their personalized learning needs [20], and thus tend to demand more teacher-learner interaction during online sessions.

There are also several important strengths of this study that are worth noting. First, a nationally representative, large-scale survey that covered nearly 25% of all the medical undergraduate students in China allows us to accurately grasp the identical characteristics of the current progress of online learning and how those are perceived by medical students. Second, our implementation of the survey was faced against challenges from the uncertainty disease outbreak and its progression. Fast design and internal validation before its official release for the survey provide valuable opportunities to capture useful information related to our topic at a critical time point. Third, despite differences in launching formal online learning among various medical education programs, our survey was being distributed almost 2 weeks after the MOE’s announcement to demand universities taking on online educational approaches, and ended the entry of survey more than 1 month after the announcement, it is suggested that our respondents are representative of medical students who have enough experience regarding online-learning since the COVID-19 outbreak. Last but not least, despite the cultural and language differences between China and many other parts of the world, the general medical education background and the implementation of online and other technology-enhanced learning between China and the rest of the world possess many similarities. The national data we collected within China provide valuable comparator and reference to the international community.

We have to acknowledge that there are a few limitations to this study. This is a cross-sectional survey conducted at the early stage of online learning implementation in various institutions in response to COVID-19 in China. We have not yet captured longitudinal data regarding the progress trajectory of these fast-iterating online-learning programs in this study. Moreover, given the nature of our survey design, we only looked at students’ perspectives towards online learning, which speaks to only Kirkpatrick Level 1, yet is crucial to the general quality evaluation; however, satisfaction itself does not measure learning as there might be potential self-report bias [35, 36]. We have not yet focused our study on the higher levels of Kirkpatrick Model, product utility, cost-effectiveness, which were frequently explored about e-learning alongside student satisfaction [37]. Another issue that our current study hasn’t touched on is the faculty and institutional perspective towards online learning, although requested by the government, as well as certain ethical concerns of confidentiality, psychological safety of the synchronized online learning environment, and the concerns regarding potentially breaching patient privacy during a more advanced learning stage among the medical students. These will the primary focus of our future studies as the COVID-19 outbreak is expected to last. Nevertheless, our study has already pointed out important directions for further research into online learning that led to student’s improved acceptance, and possible establishment of quality control and improvement criteria for online-learning as a formal educational approach in medical education. It is also predictable that issues and challenges, as well as students’ perspectives towards online learning, which have been described in our current study may mitigate overtime; certain issues could be overcome with technology advancement, and new challenges may emerge. Therefore, this study is also worth follow-up to investigate the dynamic evolution of such formal online learning and how students respond to such progression.

## Conclusions

This study presents a glimpse towards the role of online learning as a formal teaching/learning approach in medical education, as well as probes for possible directions, in a categorized manner, to improve online learning in medical education and what to maintain as a good practice, especially from the lenses of our medical students in China. The practice from China medical education could provide valuable lessons on better preparing online education for future doctors worldwide. Considerations should be made regarding such applications in aspects of students’ different learning phases. Online learning possesses great potential in promoting standardization towards medical education. This study also reveals the critical role of students’ prior online learning experience in their acceptance of new online experiences in learning about medicine. This finding implies that medical educators and administrators would put much emphasis on students’ online experiences early on. Measures should be taken from technological, organizational, environmental, as well as individual aspects, to help improve the outcome of online learning. Technological considerations and enhancement, organizational support in faculty training on both overall instructional skill and online teaching and providing better social environment could all possibly lead to better development for online learning.

## Data Availability

The English version of datasets generated and/or analyzed during the current study available from the corresponding author on reasonable request.

## Abbreviations

HCWs: Healthcare workers
MOE: Ministry of Education
NCHPED: National Center for Health Professionals Education Development
NHC: National Health Commission
TAM: Technology Acceptance Model
PU: Perceived usefulness
CMSS: China Medical Student Survey.
